# The **“**Goldilocks Zone” of immune balance: association of neutrophil-to-lymphocyte and lymphocyte-to-monocyte ratios with miscarriage

**DOI:** 10.3389/fgwh.2026.1775954

**Published:** 2026-07-13

**Authors:** Zhou Fang, Nan Ding, Fang Wang

**Affiliations:** 1 Reproductive Medicine Center, The Second Affiliated Hospital of Shandong University of Traditional Chinese Medicine, Jinan City, Shandong, China; 2 Reproductive Medicine Center, The Second Hospital of Lanzhou University, Lanzhou City, Gansu, China

**Keywords:** biomarkers, inflammation, lymphocyte-to-monocyte ratio, miscarriage, neutrophil-to-lymphocyte ratio

## Abstract

**Background:**

Miscarriage affects 15%–20% of clinically recognized pregnancies, yet reliable biomarkers for risk stratification remain elusive. While systemic inflammatory markers derived from routine complete blood count have shown promise in identifying associations with adverse pregnancy outcomes, their association with miscarriage has not been systematically evaluated.

**Methods:**

In this retrospective cohort study of 347 pregnant women with a history of at least one prior pregnancy loss, we calculated neutrophil-to-lymphocyte ratio (NLR), lymphocyte-to-monocyte ratio (LMR), and platelet-to-lymphocyte ratio (PLR) from routine complete blood count tests. Associations with miscarriage were evaluated using four progressively adjusted logistic regression models accounting for demographic, reproductive, and metabolic confounders. Dose-response relationships were assessed through quartile analysis, threshold effects through piecewise linear regression, and non-linear associations through generalized additive models.

**Results:**

After comprehensive adjustment, higher NLR was independently associated with reduced miscarriage risk (OR = 0.67 per doubling, 95%CI: 0.49–0.91, *P* = 0.012), with women in the highest quartile showing 60% lower risk than those in the lowest quartile (Q4 vs. Q1: OR = 0.398, *P* = 0.006). This protective effect was most pronounced at NLR values <4.16 (OR = 0.64, *P* = 0.001), plateauing at higher levels. Conversely, elevated LMR significantly increased miscarriage risk (OR = 1.839 per doubling, 95%CI: 1.12–3.01, *P* = 0.015), with risk more than doubling in the highest quartile (OR = 2.236, *P* = 0.017) and accelerating above LMR values of 6.46 (OR = 1.62, *P* = 0.049). No significant association was observed between PLR and pregnancy outcomes in our study.

**Conclusions:**

Among women with a history of prior pregnancy loss, NLR and LMR were independently associated with miscarriage in opposing directions, consistent with a **“**Goldilocks” conceptual framework of immune balance in early pregnancy. No significant association was observed between PLR and pregnancy outcomes in our study. These readily available CBC-derived inflammatory indices may provide useful insight into immune-related changes associated with miscarriage risk, but require prospective validation in independent cohorts.

## Introduction

Miscarriage, defined as pregnancy loss before 24 weeks of gestation, represents the most common complication of pregnancy, affecting 15%–20% of clinically recognized pregnancies and up to 26% when including biochemical pregnancies (1, 2). The majority occur during the first trimester, with profound physical and psychological impacts on affected women and their families (3, 4). Despite its high prevalence, the ability to predict and prevent miscarriage remains limited, partly due to incomplete understanding of underlying mechanisms and lack of reliable biomarkers (5).

Current predictive approaches rely primarily on ultrasonographic assessment and serum biomarkers such as human chorionic gonadotropin (hCG) and progesterone (6). However, these markers have limited sensitivity and specificity, particularly in asymptomatic women without vaginal bleeding (7). The identification of novel, accessible biomarkers associated with miscarriage risk would improve our understanding of immune-related mechanisms, particularly among women with a history of prior pregnancy loss.

Successful pregnancy requires a delicate balance of immune responses at the maternal-fetal interface (8, 9). During early pregnancy, controlled inflammatory processes are essential for embryo implantation, trophoblast invasion, and placental development (10, 11). However, dysregulated inflammation, whether excessive or insufficient, has been implicated in pregnancy complications including miscarriage, preeclampsia, and preterm birth (12, 13). The immune system undergoes dynamic changes throughout pregnancy. Early gestation is characterized by a pro-inflammatory state that facilitates implantation and placentation (14). This is followed by an anti-inflammatory period during mid-pregnancy to accommodate fetal growth, and a return to pro-inflammatory status at term to initiate labor (15). Disruption of this finely tuned inflammatory trajectory may contribute to pregnancy loss (16, 17).

Routine complete blood count (CBC) parameters have emerged as readily available indicators of systemic inflammation (18, 19). Derived ratios combining different leukocyte populations have demonstrated prognostic value across diverse clinical conditions, from cardiovascular disease to cancer (20, 21). Among these, the neutrophil-to-lymphocyte ratio (NLR), lymphocyte-to-monocyte ratio (LMR), and platelet-to-lymphocyte ratio (PLR) have garnered particular attention as markers of immune balance and inflammatory burden (22, 23).

The neutrophil-to-lymphocyte ratio reflects the balance between innate inflammatory response (neutrophils) and adaptive immune regulation (lymphocytes) (24). Elevated NLR has been associated with adverse outcomes in various pathological conditions, suggesting a role for excessive inflammation (25). However, its significance in early pregnancy remains controversial, with conflicting findings across studies (26, 27). In this study, we hypothesized that inflammatory markers would be independently associated with miscarriage risk and that these associations would demonstrate non-linear patterns consistent with the concept of optimal immune balance in pregnancy.

## Methods

### Study design and setting

This retrospective cohort study was conducted at Lanzhou University Second Hospital between September 2019 and February 2022. The study received approval from the ethical committee of Lanzhou University Second Hospital (2019A-231), and was conducted in accordance with the Declaration of Helsinki. Given the retrospective nature of the study using de-identified data from routine clinical care, the requirement for informed consent was waived by the ethics committee.

### Study population

Pregnant women were eligible for inclusion if they met the following criteria: (1) singleton pregnancy confirmed by ultrasound; (2) first prenatal visit at gestational age ≤10 weeks (calculated from last menstrual period and confirmed by ultrasound measurements); (3) a history of at least one prior pregnancy loss; (4) complete blood count (CBC) testing performed at first prenatal visit before any interventions; (5) complete clinical and demographic data available in electronic medical records; and (6) documented pregnancy outcome classified as either live birth (delivery of a viable infant at ≥24 weeks gestation) or miscarriage (spontaneous pregnancy loss before 24 weeks gestation). Women were excluded if they had: (1) elective pregnancy termination; (2) known chromosomal abnormalities or major fetal structural anomalies; (3) active infectious diseases at the time of blood sampling (including acute upper respiratory infection, urinary tract infection, or systemic infection); (4) active vaginal bleeding, acute pelvic pain, or clinical diagnosis of threatened abortion at the time of blood sampling; (5) chronic inflammatory or autoimmune conditions (including systemic lupus erythematosus, rheumatoid arthritis, inflammatory bowel disease); (6) malignancy; (7) current use of immunosuppressive medications or corticosteroids; (8) hematological disorders (including severe anemia requiring treatment, thrombocytopenia, leukemia, or other blood cell disorders); or (9) incomplete blood count data or missing covariate information.

### Blood sample collection

Blood samples were collected at the first prenatal visit at gestational age ≤10 weeks in all participants. For women who subsequently experienced miscarriage, sampling therefore preceded ultrasound confirmation of miscarriage prior to clinical outcome determination.

### Sample size calculation

Although this was a retrospective study, we performed a *post-hoc* power analysis to assess the adequacy of our sample size. Using G*Power 3.1 with logistic regression, two-tailed *z*-test, an observed odds ratio of 0.67, an event rate of 0.493, and *α* = 0.05, our final sample of 347 participants yielded an actual power of 0.951.

### Data collection

#### Clinical, demographic variables and laboratory measurements

Data were systematically extracted from electronic medical records by two trained research assistants using a standardized data collection form. The following variables were collected: maternal age at conception, pre-pregnancy body mass index (BMI, calculated as weight in kilograms divided by height in meters squared), education level (categorized as primary/junior high school, high school, or college/university and above), ethnicity (Han Chinese or ethnic minority), menstrual cycle regularity (regular cycle defined as 21–35 days or irregular cycle), reproductive history including parity, number of previous miscarriages (categorized as 0, 1, or ≥2), and pregnancy loss status (classified as primary pregnancy loss, secondary pregnancy loss). Complete blood count parameters were measured using an automated haematology analyser (Mindray BC-6800Plus, Mindray Bio-Medical Electronics Co., Ltd., Shenzhen, China) as part of routine prenatal care. Blood samples were collected via venipuncture into EDTA-containing tubes after an overnight fast or at least 4 h of fasting. Samples were processed within 2 h of collection according to standard laboratory protocols. The following parameters were recorded: white blood cell count, absolute neutrophil count (NE, ×10^9^/L), absolute lymphocyte count (LY, ×10^9^/L), absolute monocyte count (MO, ×10^9^/L), and platelet count (PLT, ×10^9^/L). Three inflammatory ratios were calculated: neutrophil-to-lymphocyte ratio (NLR = NE/LY), lymphocyte-to-monocyte ratio (LMR = LY/MO), and platelet-to-lymphocyte ratio (PLR = PLT/LY). The laboratory participates in external quality assurance programs and maintains internal quality control procedures. For participants with multiple CBC tests during early pregnancy, the earliest measurement before pregnancy outcome was used for analysis. Serum lipid profiles were also extracted from medical records, including total cholesterol (CHO, mmol/L), triglycerides (TG, mmol/L), high-density lipoprotein cholesterol (HDL, mmol/L), and low-density lipoprotein cholesterol (LDL, mmol/L). Lipid measurements were performed using enzymatic colorimetric methods on an automated biochemistry analyzer (Cobas 8000, Roche Diagnostics, Basel, Switzerland).

### Gestational Age determination and outcome ascertainment

Gestational age was determined based on the first day of the last menstrual period when the date was certain and consistent with first-trimester ultrasound findings. When menstrual dating was uncertain or discrepant, ultrasound measurements (crown-rump length or biparietal diameter) were used to establish gestational age. Pregnancy outcomes were ascertained through review of medical records, including delivery records, ultrasound reports, and clinical notes. Miscarriage was defined as spontaneous pregnancy loss before 24 completed weeks of gestation, confirmed by ultrasound showing absence of fetal cardiac activity or passage of products of conception. Live birth was defined as delivery of a viable infant at or after 24 weeks gestation with signs of life.

### Statistical analysis

Baseline characteristics were summarized using appropriate descriptive statistics according to variable distribution. Continuous variables are presented as mean ± standard deviation or median (interquartile range, IQR), and categorical variables as counts (percentages). Between-group comparisons were performed using Student's *t*-test or Mann–Whitney *U*-test for continuous variables and chi-square or Fisher's exact test for categorical variables, as appropriate.

Neutrophil-to-lymphocyte ratio (NLR), lymphocyte-to-monocyte ratio (LMR), and platelet-to-lymphocyte ratio (PLR) showed right-skewed distributions and were therefore log_2_-transformed prior to regression analyses, allowing effect estimates to be interpreted as the change in odds associated with a doubling of marker levels. Associations between inflammatory markers and miscarriage were evaluated using multivariable logistic regression with four progressively adjusted models, including demographic characteristics, reproductive history, lifestyle-related factors, and lipid profiles. Odds ratios (ORs) with 95% confidence intervals (CIs) and two-sided *p* values were reported.

Dose–response relationships were further examined by categorizing inflammatory markers into quartiles, with the lowest quartile as the reference group, and linear trend was assessed by modeling quartiles as ordinal variables. Potential non-linear associations were explored using generalized additive models (GAMs) adjusted for the fully adjusted model covariates. Based on observed non-linear patterns, threshold effects were evaluated using piecewise linear regression with inflection points identified by profile likelihood methods. All analyses were performed using R software (version 4.3.1), and *p* values <0.05 were considered statistically significant.

## Results

### Study population and baseline characteristics

During the study period, 428 pregnant women with singleton pregnancies and a history of at least one prior pregnancy loss were initially assessed for eligibility. After application of the exclusion criteria, 81 women were excluded, including those with elective pregnancy termination (approximately *n* = 15), chronic inflammatory or autoimmune diseases (*n* = 29), use of immunosuppressive medications or corticosteroids (*n* = 7), and incomplete blood count data or missing covariate information (*n* = 30). After exclusions, a total of 347 women were included in the final analysis, of whom 176 (50.7%) had live births and 171 (49.3%) experienced miscarriages (Table 1). The two groups were comparable in terms of maternal age (median 31 vs. 30 years, *P* = 0.72), body mass index (*P* = 0.178), education level (*P* = 0.697), ethnicity (*P* = 0.899), menstrual regularity (*P* = 0.29), and infertility status (*P* = 0.995). As expected, women with miscarriage had a higher proportion of previous pregnancy losses (≥3 losses: 26.9% vs. 14.2%, *P* = 0.002), confirming this as a known risk factor. Regarding metabolic parameters, high-density lipoprotein cholesterol was slightly lower in the miscarriage group (1.2 vs. 1.3 mmol/L, *P* = 0.028), while total cholesterol, triglycerides, and low-density lipoprotein showed no significant differences between groups. Importantly, baseline inflammatory parameters differed significantly between groups. Women with live births had higher median neutrophil counts (5.4 vs. 4.4 × 10^9^/L, *P* < 0.001) and higher neutrophil-to-lymphocyte ratio (3.2 vs. 2.8, *P* = 0.001), while monocyte counts were also higher (0.4 vs. 0.3 × 10^9^/L, *P* = 0.018). Conversely, the lymphocyte-to-monocyte ratio was significantly lower in the live birth group (4.6 vs. 5.1, *P* = 0.003). Platelet counts and platelet-to-lymphocyte ratio showed no significant differences (both *P* > 0.4).

**Table 1:** Baseline characteristics of study population stratified by pregnancy outcome.

Variables	Total (*n* = 347)	Live birth (*n* = 176)	Miscarriage (*n* = 171)	*p*
Age,Year, Median (Q1,Q3)	30 (27, 33)	31 (28, 33)	30 (27, 33)	0.72
BMI, (kg/m^2^), Median (Q1,Q3)	22.7 (20.7, 24.8)	22 (20.5, 24.5)	23.1 (20.8, 24.8)	0.178
Education (%)				0.697
Primary/Junior	49 (14.1)	27 (15.3)	22 (12.9)	
High school	76 (21.9)	40 (22.7)	36 (21.1)	
College or above	222 (64)	109 (61.9)	113 (66.1)	
Ethnicity, *n* (%)				0.899
Han Chinese	321 (92.5)	162 (92)	159 (93)	
other ethnicities	26 (7.5)	14 (8)	12 (7)	
Menstrual cycle, *n* (%)				0.29
Irregular	273 (78.7)	143 (81.2)	130 (76)	
Regularity	74 (21.3)	33 (18.8)	41 (24)	
Prior pregnancy losses (%)				0.002
1	162 (46.7)	97 (55.1)	65 (38)	
2	114 (32.9)	54 (30.7)	60 (35.1)	
≥3	71 (20.5)	25 (14.2)	46 (26.9)	
Type of pregnancy loss				0.995
Primary	281 (81)	142 (80.7)	139 (81.3)	
Secondary	66 (19)	34 (19.3)	32 (18.7)	
CHO, mmol/L,Median (Q1,Q3)	3.7 (3.2, 4.2)	3.7 (3.3, 4.3)	3.6 (3.1, 4.2)	0.058
TG, mmol/L,Median (Q1,Q3)	1 (0.8, 1.5)	1.1 (0.7, 1.5)	1 (0.8, 1.4)	0.902
HDL, mmol/L, Median (Q1,Q3)	1.3 (1.1, 1.5)	1.3 (1.1, 1.6)	1.2 (1.1, 1.5)	0.028
LDL, mmol/L, Median (Q1,Q3)	2.3 (1.9, 2.7)	2.3 (1.9, 2.8)	2.2 (1.9, 2.7)	0.367
NE, 10^9^/L, Median (Q1,Q3)	4.8 (3.8, 6.6)	5.4 (3.9, 6.9)	4.4 (3.6, 6)	< 0.001
LY,10^9^/L, Median (Q1,Q3)	1.6 (1.4, 2.1)	1.6 (1.3, 2.1)	1.7 (1.4, 2.1)	0.42
MO, 10^9^/L, Median (Q1,Q3)	0.3 (0.3, 0.4)	0.4 (0.3, 0.4)	0.3 (0.2, 0.4)	0.018
PLT, 10^9^/L, Mean ± SD	231.7 ± 66.1	229.2 ± 61.5	234.4 ± 70.7	0.47
NLR, Median (Q1,Q3)	3 (2.2, 4.1)	3.2 (2.3, 4.4)	2.8 (2, 3.5)	0.001
LMR, Median (Q1,Q3)	4.8 (3.7, 6)	4.6 (3.6, 5.6)	5.1 (4, 6.5)	0.003
PLR, Median (Q1,Q3)	138.5 (109, 170.5)	137.3 (109, 171)	138.5 (108.8, 169.6)	0.866

### Multivariable logistic regression analysis

Table 2 presents the associations between inflammatory markers and miscarriage risk across four progressively adjusted models, analyzing both continuous variables (per log2 increase) and categorical variables (quartiles) to comprehensively assess dose-response relationships. For NLR analyzed as a continuous variable, a consistent protective association was observed across all models. In the crude model (Model 1), each doubling of NLR was associated with a 35% reduction in miscarriage odds (OR = 0.651, 95% CI: 0.484–0.876, *P* = 0.005). This association strengthened slightly after adjusting for maternal age and previous miscarriage history (Model 2: OR = 0.635, 95% CI: 0.469–0.859, *P* = 0.003), remained stable after additional adjustment for demographic factors (Model 3: OR = 0.638, 95% CI: 0.468–0.869, *P* = 0.004), and persisted in the fully adjusted model including lipid profile (Model 4: OR = 0.670, 95% CI: 0.491–0.914, *P* = 0.012). Quartile analysis revealed a clear dose-response relationship. Using the lowest quartile (Q1) as reference, women in the highest NLR quartile had approximately 60% lower odds of miscarriage (Q4 vs. Q1: OR = 0.398, 95% CI: 0.21–0.77, *P* = 0.006), with significant linear trend (P for trend = 0.005). This dose-response pattern remained consistent across all four models (P for trend = 0.0012–0.005).

**Table 2:** Multivariable logistic regression analysis of inflammatory markers and miscarriage.

Variables		Model 1 OR (95%CI)	*p-value*	Model 2 OR (95%CI)	*p-value*	Model 3 OR (95%CI)	*p-value*	Model 4 OR (95%CI)	*p-value*
Continuous log2(NLR)		0.651 (0.484–0.876)	0.005	0.635 (0.469–0.859)	0.003	0.638 (0.468–0.869)	0.004	0.67 (0.491–0.914)	0.012
Categories log2(NLR)									
	Q1	Reference		Reference		Reference		Reference	
	Q2	0.625 (0.34–1.14)	0.127	0.648 (0.35–1.20)	0.167	0.648 (0.35–1.21)	0.172	0.618 (0.33–1.17)	0.138
	Q3	0.507 (0.28–0.93)	0.028	0.523 (0.28–0.97)	0.04	0.522 (0.28–0.98)	0.043	0.51 (0.27–0.97)	0.039
	Q4	0.392 (0.21–0.72)	0.003	0.36 (0.19–0.68)	0.001	0.361 (0.19–0.69)	0.002	0.398 (0.21–0.77)	0.006
	*P* for trend		0.0021		0.0012		0.0016		0.005
Continuous log2(LMR)		2.012 (1.266–3.196)	0.003	2.006 (1.25–3.222)	0.004	1.955 (1.204–3.173)	0.007	1.839 (1.123–3.01)	0.015
Categories log2(LMR)									
	Q1	Reference		Reference		Reference		Reference	
	Q2	1.455 (0.80–2.66)	0.222	1.497 (0.81–2.77)	0.2	1.553 (0.82–2.92)	0.173	1.417 (0.74–2.71)	0.291
	Q3	1.488 (0.81–2.72)	0.197	1.537 (0.83–2.85)	0.173	1.454 (0.77–2.74)	0.247	1.318 (0.69–2.52)	0.403
	Q4	2.43 (1.32–4.47)	0.004	2.46 (1.32–4.60)	0.005	2.487 (1.30–4.74)	0.006	2.236 (1.16–4.32)	0.017
	*P* for trend		0.0061		0.0067		0.0102		0.0272
Continuous log2(PLR)		0.977 (0.648–1.472)	0.91	1.018 (0.67–1.548)	0.932	0.995 (0.647–1.53)	0.982	0.993 (0.63–1.563)	0.974
Categories log2(PLR)									
	Q1	Reference		Reference		Reference		Reference	
	Q2	0.955 (0.53–1.73)	0.879	0.883 (0.48–1.62)	0.688	0.792 (0.42–1.48)	0.465	0.74 (0.39–1.41)	0.358
	Q3	1.072 (0.59–1.95)	0.819	1.007 (0.55–1.85)	0.981	1.006 (0.54–1.87)	0.984	0.962 (0.51–1.82)	0.905
	Q4	0.955 (0.53–1.73)	0.879	1.004 (0.55–1.84)	0.991	0.976 (0.53–1.81)	0.939	1.031 (0.55–1.94)	0.925
	*P* for trend		0.9806		0.8859		0.8723		0.7301

OR, odds ratio; CI, confidence interval.

Model 1 was the crude model without covariate adjustment.

Model 2 was adjusted for age and number of previous pregnancy losses.

Model 3 was further adjusted for body mass index (BMI), education level, ethnicity, menstrual regularity, and primary/secondary infertility status.

Model 4 was additionally adjusted for lipid parameters, including total cholesterol (CHO), triglycerides (TG), high-density lipoprotein cholesterol (HDL), and low-density lipoprotein cholesterol (LDL).

In categorical analyses, the lowest quartile (Q1) was used as the reference category.

P for trend was calculated by modeling quartile categories as an ordinal variable in the multivariable logistic regression models.

A two-sided *P* value < 0.05 was considered statistically significant.

In contrast to NLR, elevated LMR was consistently associated with increased miscarriage risk across all models. Each doubling of LMR increased the odds of miscarriage by 101% in the crude model (OR = 2.012, 95% CI: 1.266–3.196, *P* = 0.003), and this adverse association persisted after full adjustment (Model 4: OR = 1.839, 95% CI: 1.123–3.01, *P* = 0.015). Quartile analysis demonstrated a progressive increase in miscarriage risk with higher LMR. Women in the highest LMR quartile had more than twice the odds of miscarriage compared to those in the lowest quartile (Q4 vs. Q1: OR = 2.236, 95% CI: 1.16–4.32, *P* = 0.017), with significant linear trend in Models 1–3 (P for trend = 0.006–0.010) that approached significance in Model 4 (P for trend = 0.027). The attenuation of the LMR association from Model 1 to Model 4 suggests that some of the LMR effect may be mediated through lipid metabolism, though the association remained independently significant after full adjustment.

Unlike NLR and LMR, no statistically significant association was observed between PLR and miscarriage risk in our study. Both continuous analysis (Model 4: OR = 0.993, 95% CI: 0.63–1.563, *P* = 0.974) and categorical analysis (Q4 vs. Q1: OR = 1.031, 95% CI: 0.55–1.94, *P* = 0.925) demonstrated no relationship, with consistently non-significant P for trend values (*P* > 0.7) across all models.

### Non-linear relationships and threshold effects

**Figure 1:**
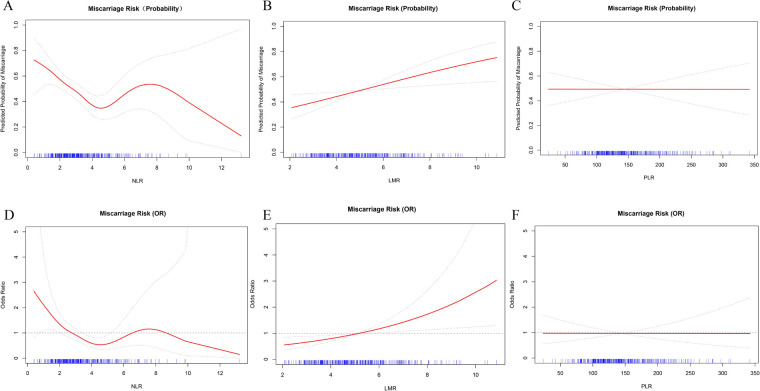
Generalized additive model (GAM) analysis of inflammatory markers and miscarriage risk. Generalized additive model (GAM) smoothing curves showing associations between inflammatory markers (NLR, LMR, PLR) and miscarriage risk. Upper panels **(A–C)**: Predicted probability of miscarriage. Lower panels **(D–F)**: Odds ratios with median as reference (OR = 1.0). Red lines indicate fitted curves; gray shaded areas show 95% confidence intervals. Models adjusted for maternal age, previous miscarriage, BMI, education, ethnicity, menstrual regularity, infertility status, and lipid profile.

To explore potential non-linear associations and identify clinically actionable cutoff values, we performed generalized additive model (GAM) analyses (Figure 1) followed by piecewise linear regression to detect threshold effects (Table 3). The GAM curve for NLR revealed a steep protective gradient at lower values that plateaus at moderate to high levels. The predicted probability of miscarriage decreased sharply from approximately 0.70 at very low NLR values (<2) to approximately 0.35 at moderate NLR values (3–5), then remained relatively stable at higher levels (Figure 1A). Correspondingly, the odds ratio decreased from >2.5 at very low NLR to approximately 0.6 at NLR values of 3–5, with a plateau thereafter (Figure 1D). This non-linear pattern suggested the presence of a threshold effect, which was confirmed by piecewise linear regression identifying an optimal inflection point at 4.16 (Table 3). Below this threshold, higher NLR was significantly protective against miscarriage (OR = 0.64, 95% CI: 0.50–0.84, *P* = 0.001), indicating that each unit increase in NLR below 4.16 was associated with a 36% reduction in miscarriage odds. However, above this threshold, the protective effect disappeared (OR = 1.15, 95% CI: 0.90–1.47, *P* = 0.268), confirming the plateau effect observed in the GAM curve. The wide confidence intervals at extreme NLR values in the GAM reflect the smaller number of observations in these ranges, but the overall trend is clear and consistent with biological plausibility.

**Table 3:** Threshold effect analysis of inflammatory markers using piecewise linear regression.

**Marker**	**Inflection point (K)**	**Segment**	**OR (95% CI)**	***P* value**
NLR	4.16	< 4.16	0.64 (0.50–0.84)	0.001
		≥ 4.16	1.15 (0.90–1.47)	0.268
LMR	6.46	< 6.46	1.10 (0.89–1.35)	0.385
		≥ 6.46	1.62 (1.00–2.62)	0.049
PLR	126.94	< 126.94	1.00 (0.98–1.01)	0.530
		≥ 126.94	1.00 (0.99–1.01)	0.653

Inflection points were identified by profile likelihood methods. Odds ratios (ORs) and 95% confidence intervals (CIs) were estimated separately for marker levels below and above the inflection point. Models were adjusted for age, number of previous pregnancy losses, body mass index, education level, ethnicity, menstrual regularity, infertility type, and lipid profiles.

The GAM curve for LMR showed a progressive, approximately linear increase in risk across its range. The predicted probability of miscarriage remained relatively stable at approximately 0.40 for LMR values below 5–6, then increased notably to >0.60 for LMR values above 7–8 (Figure 1B). The corresponding odds ratio curve demonstrated a steady increase from approximately 0.6 at low LMR values to >2.5 at high values (Figure 1E). Piecewise linear regression identified a threshold at 6.46, below which LMR showed no significant association with miscarriage (OR = 1.10, 95% CI: 0.89–1.35, *P* = 0.385), but above which each unit increase in LMR was associated with significantly increased miscarriage risk (OR = 1.62, 95% CI: 1.00–2.62, *P* = 0.049). This pattern confirms that LMR becomes a risk factor only when it exceeds this threshold, with risk accelerating above this point. Unlike the plateau observed for NLR, the LMR risk curve continues to rise without apparent ceiling, suggesting that higher lymphocyte predominance translates to progressively greater miscarriage risk.

Consistent with all previous analyses, the GAM curves for PLR were essentially flat. The predicted probability oscillated around 0.45–0.50 across the entire range of PLR values with no clear trend (Figure 1C), and the odds ratio curve fluctuated around 1.0 with wide confidence bands (Figure 1F). Piecewise linear regression confirmed the absence of threshold effects, with no significant associations either below or above the inflection point at 126.94 (both *P* > 0.5, Table 3). The lack of association for PLR across all analytical approaches (continuous, categorical, non-linear, and threshold analyses) strengthens confidence in the specificity of the NLR and LMR findings, suggesting that not all CBC-derived inflammatory markers are relevant to early pregnancy loss and that the associations observed for NLR and LMR are unlikely to be artifacts of multiple testing or spurious correlations.

## Discussion

In this retrospective cohort study of 347 pregnant women with a history of prior pregnancy loss, we identified neutrophil-to-lymphocyte ratio (NLR) and lymphocyte-to-monocyte ratio (LMR) as independently associated markers of early pregnancy loss with opposing effects, while platelet-to-lymphocyte ratio (PLR) showed no association. Three key findings emerged. First, higher NLR was associated with a reduced risk of miscarriage, with each doubling in NLR corresponding to a 33% decrease in adjusted odds of pregnancy loss. This association demonstrated a clear dose–response pattern and a threshold effect, with the protective association most evident below an NLR value of 4.16. Second, elevated LMR was independently associated with increased miscarriage risk, showing both a dose–response relationship and a threshold effect above 6.46. To our knowledge, this is the first study to identify LMR as a risk marker for miscarriage. Third, the absence of association between PLR and miscarriage strengthens the specificity of our findings for NLR and LMR rather than reflecting a nonspecific inflammatory signal.

Our finding that higher NLR is associated with reduced miscarriage risk aligns with some but not all previous studies, highlighting the complexity and context-dependency of inflammatory markers in pregnancy. Kim et al. reported that NLR was significantly lower in women with missed abortion compared to those with threatened abortion, suggesting that higher NLR may be associated with a reduced likelihood of progression to missed abortion, consistent with our findings (28). In contrast, Turgut et al. and several other studies found elevated inflammatory markers in women with miscarriage (27). This apparent contradiction can be explained by critical differences in study design, particularly the timing of blood sampling. Studies reporting elevated inflammatory markers in miscarriage typically collected samples at or after the time of miscarriage diagnosis, thereby capturing inflammatory responses secondary to pregnancy loss rather than predictive baseline status. Our study, along with Kim's, sampled women during routine early pregnancy care before pregnancy outcome was known, potentially detecting baseline inflammatory status that is associated with subsequent outcomes rather than consequences of miscarriage.

The identification of LMR as an independent risk factor for miscarriage represents a novel contribution. While NLR has been extensively studied in pregnancy complications including preeclampsia, preterm birth, and gestational diabetes (29–31), LMR has received far less attention in obstetric literature. To our knowledge, this is the first study to demonstrate a dose-response relationship between elevated LMR and miscarriage risk, identify a specific threshold (6.46), and show that this association persists after comprehensive adjustment for demographic and metabolic confounders. This finding raises new questions about the potential role of immune dysregulation in pregnancy loss.

The opposing associations observed for NLR and LMR are consistent with a “Goldilocks” conceptual framework of immune balance in early pregnancy, whereby successful pregnancy may require a balanced immune state that is neither insufficient nor excessive (8, 9). We propose this as a working hypothesis, as peripheral blood CBC ratios are indirect proxies for complex local immune processes. This concept is consistent with established understanding of pregnancy immunology, where successful pregnancy requires precise orchestration of both innate and adaptive immunity (14). Moderately elevated NLR may reflect a degree of innate immune activation that is associated with favourable pregnancy outcomes, possibly through mechanisms related to decidual preparation and early placentation. Neutrophils are not merely passive inflammatory cells but active participants in early pregnancy establishment. Neutrophils are known to participate in early pregnancy establishment through multiple pathways, and adequate neutrophil activity has been proposed to support implantation-related processes (32, 33). However, whether the NLR associations observed in peripheral blood reflect these local decidual processes remains speculative and requires direct investigation. In contrast, elevated LMR may be associated with alterations in adaptive immune balance at the maternal-fetal interface. During normal pregnancy, the maternal immune system must achieve a delicate balance: maintaining tolerance to paternal antigens while preserving the ability to defend against infections. This is accomplished through complex mechanisms including regulatory T cell expansion, skewing from pro-inflammatory Th1 to anti-inflammatory Th2 responses, and carefully controlled decidual natural killer cell activation (34, 35). We hypothesise that elevated LMR, reflecting relative lymphocyte predominance, may be associated with shifts in this balance that are less favourable for pregnancy maintenance. However, the specific cellular and molecular mechanisms underlying this association cannot be determined from peripheral blood ratios alone, pending confirmation through studies incorporating direct immunological assessment. The observation that miscarriage risk appears to accelerate above an LMR of 6.46 is consistent with the possibility of a threshold beyond which these immune shifts become clinically relevant.

From a clinical perspective, the identified thresholds for NLR and LMR offer potential utility for early risk stratification using routinely available complete blood count data. These markers are inexpensive, widely accessible, and already incorporated into standard prenatal care, making them attractive candidates for risk assessment, particularly in settings where specialized immunological testing is unavailable. Women with a history of prior pregnancy loss who exhibit high-risk inflammatory profiles could benefit from closer monitoring and counselling in early pregnancy. However, these findings should not yet be used to guide clinical interventions, as their clinical utility requires prospective validation in independent cohorts.

## Conclusions

In conclusion, we identified NLR and LMR as independently associated markers of early pregnancy loss with opposing effects, while PLR showed no association. Higher NLR was protective, whereas elevated LMR increased miscarriage risk, with clinically relevant threshold effects observed for both markers. These findings support the concept that a balanced immune state is essential for early pregnancy success. As readily available markers derived from routine blood tests, NLR and LMR hold promise for early risk stratification among women with a history of prior pregnancy loss, although prospective validation in independent cohorts remains essential before implementation in practice.

### Strengths and limitations

This study has several strengths. We employed a comprehensive analytical strategy incorporating progressive multivariable adjustment, dose–response assessment using quartiles, threshold detection, and non-linear visualization, providing consistent evidence across multiple approaches. We also adjusted for a broad range of demographic, reproductive, and metabolic confounders, enhancing the robustness of our findings.

Several limitations should be acknowledged. The retrospective observational design precludes causal inference, and residual confounding by unmeasured factors cannot be excluded. Inflammatory markers were measured at a single time point, preventing assessment of temporal changes during early pregnancy. The single-centre nature of the study and the restriction to women with a history of prior pregnancy loss may limit generalisability, underscoring the need for external validation in independent, multi-centre cohorts. Finally, we lacked detailed immunophenotyping data, including peripheral NK cell quantification, lymphocyte subset profiling, and cytokine measurements, to directly elucidate the underlying biological mechanisms linking CBC-derived inflammatory indices to pregnancy outcomes.

## Data Availability

The raw data supporting the conclusions of this article will be made available by the authors, without undue reservation.
